# Parental Attitude, Knowledge, and Practices Regarding the Usage of Antibiotics for Upper Respiratory Tract Infections in Children During the COVID-19 Pandemic

**DOI:** 10.7759/cureus.39932

**Published:** 2023-06-04

**Authors:** Kanwal Chand, Muhammad Ismail Butt, Hafiz Muhammad Tahir

**Affiliations:** 1 Department of Paediatric Medicine, Central Hospital, Delhi, IND; 2 Department of Medicine, Tehsil Headquarter Hospital (THQ) Hospital, Pasrur, PAK; 3 Department of Medicine, Tehsil Headquarter Hospital (THQ) Hospital, Lalian, PAK

**Keywords:** knowledge, behaviors, attitudes, covid-19, pandemic, upper respiratory tract infections

## Abstract

Background: The COVID-19 pandemic has raised knowledge of the proper antibiotic dosage for treating childhood upper respiratory tract infections (URTIs). In order to ensure proper antibiotic usage and prevent the establishment of illnesses that is antibiotic-resistant during the COVID-19 pandemic, parental attitudes, knowledge, and behaviour surrounding antibiotic use for URTIs in children are essential. The goal of this study was to find out the parental attitude, knowledge, and practices regarding the usage of antibiotics for URTIs in children during the COVID-19 epidemic.

Methodology: This cross-sectional was conducted in the Department of Paediatric Medicine, Central Hospital, Ganesh Nagar, New Delhi, India from September 2022 to February 2023. The study analysed a total of 500. All the children had URTIs. A structured questionnaire was randomly distributed among parents. Socio-demographic information like gender, age, occupation, monthly family income, and age of the children were noted at the time of enrollment. Outcomes were recorded in terms of responses to questions regarding attitude, knowledge, and practices regarding the use of antibiotics for URTIs in children during the COVID-19 epidemic.

Results: Of a total of 500 parents, 380 (76.0%) were male. The mean age was 39.9±8.3 years while 280 (56.0%) participants were aged between 31 to 45 years. Relatively older age (p<0.0001) and occupational status as unemployed (p<0.0001) were found to have a significant association with response to "virus being the cause of COVID-19". Females (p=0.0004) and increasing age (p<0.0001) were found to have significant associations with incorrect responses to “antibiotics are essential for managing the symptoms in children with COVID-19”. Incorrect responses to “without the use of antibiotics, children usually suffer from greater periods of sickness” were associated with females and increasing age (p<0.0001). Incorrect responses to “not using antibiotics will prove beneficial for the children suffering from COVID-19” were significantly associated with female gender (p=0.0016) and increasing age (p<0.0001). The incorrect responses to “how often are antibiotics being prescribed to the COVID-19 children” was significantly linked with females and relatively older age (p<0.0001).

Conclusions: Parental attitude, knowledge, and practices regarding the usage of antibiotics for URTIs in children during the COVID-19 epidemic showed variations. Parental attitude, knowledge, and practices were associated with gender, age, and socio-economic status.

## Introduction

Upper respiratory tract infections (URTIs) are very communal in children, particularly those under the age of six [1]. These infections are typically caused by viruses and can affect the nose, throat, sinuses, and ears. Some common types of URTIs in children include common pharyngitis, sinusitis, tonsillitis, cold, and otitis media [2]. The common cold is the most prevalent URTI in children and is caused by a range of viruses. Symptoms can include runny nose, congestion, cough, and sore throat. Sinusitis is due to infection of the air-filled sinus cavities in the face. It can cause symptoms such as nasal congestion, headache, and facial pain [3]. Pharyngitis is an infection of the throat, which can cause sore throat, fever, and difficulty in swallowing. Tonsillitis is an infection of the tonsils, which are lymph nodes at the back of the throat [4]. Symptoms can include sore throat, fever, and swollen tonsils. Otitis media is an ear infection that can result in ear pain, fever, and hearing loss [5].

The epidemiology of URTIs in children can vary depending on several factors, including age, season, and location. URTIs are most common in children under the age of six, with the highest incidence occurring in children aged one to three years [6]. This is likely due to the fact that young children are more likely to be exposed to viruses, as they have not yet developed immunity to many of them. URTIs tend to be more common in the winter months, likely due to the fact that many viruses that cause URTIs thrive in colder, drier environments [7]. However, URTIs can occur year-round. The incidence of URTIs can vary by geographic location, as some viruses that cause URTIs may be more prevalent in certain areas [8]. For example, respiratory syncytial virus (RSV) is more common in tropical climates, while influenza is more common in temperate climates. Certain factors can increase a child's risk of developing URTIs, including attending daycare, exposure to cigarette smoke, and having underlying medical conditions that weaken the immune system [9].

Treatment for URTIs in children usually involves relieving symptoms with over-the-counter medications, such as acetaminophen or ibuprofen for pain and fever, and saline nasal drops or sprays to help with congestion [10]. Antibiotics are not usually necessary, as most URTIs are caused by viruses and will resolve on their own within a week or two. However, if a bacterial infection is suspected or if symptoms do not improve after a few days, a doctor may prescribe antibiotics [11]. Strategies to prevent URTIs in children include promoting the good practice of hygiene, i.e., evading sick individuals' close contact and frequent washing of hands [12]. Vaccines are also available to protect against some viruses that cause URTIs, such as the flu vaccine and the RSV vaccine for high-risk infants [13].

It is assumed that the COVID-19 pandemic has contributed to awareness of the proper administration of antibiotics, especially when it comes to paediatric URTIs. Although viruses are the most prevalent cause of URTIs in children, medications are only effective in treating bacterial infections [14]. There have been concerns that the antibiotics misuse for the treatment of URTIs during the COVID-19 pandemic may contribute to the emergence of antibiotic-resistant illnesses, which could pose a serious threat to public health [15]. To address this concern, healthcare providers have been encouraged to follow evidence-based guidelines that recommend against the routine use of antibiotics for viral infections [16]. Instead, symptomatic relief with supportive measures, such as rest, fluids, and over-the-counter medications, is often recommended. Overall, it is important for physicians to judicially prescribe antibiotics in order to prevent the emergence of drug-resistant bacteria [17,18]. Accordingly, the goal of this study was to find out the parental attitude, knowledge, and practices regarding the usage of antibiotics for URTIs in children during the COVID-19 epidemic.

## Materials and methods

This cross-sectional study was conducted in the Department of Paediatric Medicine of Central Hospital, Ganesh Nagar, New Delhi, India from September 2022 to February 2023. Parents of children who received primary paediatric medical care and were aged up to 16 years were analysed. Parents who did not give consent to be part of this study were excluded. Parents were asked to completely respond to two separate components of an anonymous self-administered questionnaire. Part A was made up of questions about occupation, gender, age, single-family status, level of education, chronic illnesses in children, health insurance status, and access to the healthcare system. Part B was made up of questions about what people know, do, and think about the use of antibiotics for URTIs and COVID-19. The structured questionnaire used for the study consisted of six questions. Approval from the institution's Ethical Committee was obtained. The local institutional ethical committee approved the utilisation of the adopted questionnaire for this research. Socio-demographic information like gender, age, occupation, monthly family income, and age of the children were noted at the time of enrollment. Outcomes were recorded in terms of responses to questions regarding attitude, knowledge, and practices regarding the use of antibiotics for URTIs in children during the COVID-19 epidemic. 

The data were summarised for descriptive analysis based on demographic traits, attitudes, knowledge, and practices concerning antibiotics use. The chi-square test was used to scrutinise the associations between parental knowledge, attitude, practices, and major demographic factors. The statistical software Statistical Package for Social Sciences (SPSS), version 21.0 (IBM Corp. Armonk, NY) was used for data analysis by setting the level of significance to 0.05. To ensure accuracy, uncertain responses were scored as incorrect. 

## Results

In a total of 500 study participants, 380 (76.0%) were male showing a male predominance of 3.2:1. The mean age was 39.9±8.3 years while 280 (56.0%) participants were aged between 31 to 45 years. Assessment of monthly family income revealed that 250 (50.0%) participants belonged to middle socio-economic status. Table 1 shows details of the demographic characteristics of the participants.

**Table 1:** Demographic characteristics of the parents

Demographic characteristics	Frequency	Percentage
Gender		
Male	380	76.0%
Female	120	24.0%
Age of parents (years)		
15-30	60	12.0%
31-45	280	56.0%
46-60	160	32.0%
Occupation		
Employed	250	50.0%
Unemployed	45	9.0%
Part-time	60	12.0%
Full-time	115	23.0%
Student	30	6.0%
Monthly family income		
Low	115	23.0%
Middle	250	50.0%
High	135	27.0%
Age of child (years)		
0-6	135	27.0%
7-11	201	40.2%
12-16	164	32.8%

The incorrect responses to “virus being the cause of COVID-19” were found to have a significant association with relatively older age (p<0.0001) and occupation status as unemployed (p<0.0001). Females (p=0.0004) and increasing age (p<0.0001) were found to have significant associations with incorrect responses to “antibiotics are essential for managing the symptoms in children with COVID-19”. Incorrect responses to “without the use of antibiotics, children usually suffer from greater periods of sickness” were associated with females (p<0.0001) and increasing age (p<0.0001). Incorrect responses to “not using antibiotics will prove beneficial for the children suffering from COVID-19” were significantly associated with females (p=0.0016) and increasing age (p<0.0001). The incorrect response to “how often the antibiotics are being prescribed to the COVID-19 children” was significantly associated with females and relatively older age (p<0.0001) as shown in Table 2.

**Table 2:** Comparison of gender, age and occupation with respect to parental attitude, knowledge, and practices regarding the usage of antibiotics for upper respiratory tract infections in children during the COVID-19 epidemic

Question statement	Response	Gender		Age (years)			Occupation	
		Male (n=380)	Female (n=120)	15-30 (n=60)	31-45 (n=280)	46-60 (n=160)	Employed (n=250)	Unemployed (n=250)
Virus being the cause of COVID-19?	Correct (n=405)	308 (81.1%)	97 (80.8%)	46 (76.7%)	261 (93.2%)	98 (61.3%)	221 (88.4%)	184 (73.6%)
	Incorrect (n=95)	72 (18.9%)	23 (19.2%)	14 (23.3%)	19 (6.8%)	62 (38.7%)	29 (11.6%)	66 (26.4%)
	P-value	0.9574		<0.0001			<0.0001	
Cold and flu being the symptoms of COVID-19 in children?	Correct (n=355)	268 (70.5%)	87 (72.5%)	35 (58.3%)	206 (73.6%)	114 (71.3%)	183 (73.2%)	172 (68.8%)
	Incorrect (n=145)	112 (29.5%)	33 (27.5%)	25 (41.7%)	74 (26.4%)	46 (28.7%)	67 (26.8%)	78 (31.2%)
	P-value	0.6779		0.0614			0.2783	
Antibiotics are essential for managing the symptoms in children with COVID-19?	Correct (n=175)	149 (39.2%)	26 (21.7%)	32 (53.3%)	110 (39.3%)	33 (20.6%)	94 (37.6%)	81 (32.4%)
	Incorrect (n=325)	231 (60.8%)	94 (78.3%)	28 (46.7%)	170 (60.7%)	127 (79.4%)	156 (62.4%))	169 (67.6%)
	P-value	0.0004		<0.0001			0.2229	
Without the use of antibiotics, children usually suffer from greater periods of sickness?	Correct (n=195)	152 (40.0%)	43 (35.8%)	40 (66.7%)	85 (29.3%)	70 (43.8%)	104 (41.6%)	91 (36.4%)
	Incorrect (n=305)	228 (60.0%)	77 (64.2%)	20 (33.3%)	195 (70.7%)	90 (56.2%)	146 (58.4%)	159 (63.6%)
	P-value	<0.0001		<0.0001			0.2333	
Not using antibiotics will prove beneficial for the children suffering from COVID-19?	Correct (n=90)	80 (21.1%)	10 (8.3%)	32 (53.3%)	31 (11.1%)	27 (16.9%)	40 (16.0%)	50 (20.0%)
	Incorrect (n=410)	300 (78.9%)	110 (91.7%)	28 (46.7%)	249 (88.9%)	133 (83.1%)	210 (84.0%)	200 (80.0%)
	P-value	0.0016		<0.0001			0.2444	
How often are antibiotics being prescribed to the COVID-19 children?	Correct (n=360)	324 (85.3%)	36 (30.0%)	30 (50.0%)	257 (91.8%)	73 (45.6%)	174 (69.6%)	186 (74.4%)
	Incorrect (n=140)	56 (14.7%)	84 (70.0%)	30 (50.0%)	23 (8.2%)	87 (54.4%)	76 (30.4%)	64 (25.6%)
	P-value	<0.0001		<0.0001			0.2320	

The details about the comparison of age of the child and monthly family income with respect to parental attitude, knowledge, and practices regarding the usage of antibiotics for upper respiratory tract infections in children during the COVID-19 epidemic are shown in Table 3.

**Table 3:** Comparison of age of the child and monthly family income with respect to parental attitude, knowledge, and practices regarding the usage of antibiotics for upper respiratory tract infections in children during the COVID-19 pandemic

Question statement	Response	Age of the child (years)			Monthly Family Income		
		0-6 (n=135)	7-11 (n=201)	12-16 (n=164)	Low (n=115)	Middle (n=250)	High (n=135)
Virus being the cause of COVID-19?	Correct (n=405)	113 (83.7%)	152 (75.6%)	140 (85.4%)	91 (79.1%)	196 (78.4%)	118 (87.4%)
	Incorrect (n=95)	22 (16.3%)	49 (24.4%)	24 (14.6%)	24 (20.9%)	54 (21.6%)	17 (12.6%)
	P-value	0.0397			0.0837		
Cold and flu being the symptoms of COVID-19 in children?	Correct (n=355)	104 (77.0%)	138 (68.7%)	113 (68.9%)	82 (71.3%)	172 (68.8%)	101 (74.8%)
	Incorrect (n=145)	31 (23.0%)	63 (31.3%)	51 (31.1%)	33 (28.7%)	78 (31.2%)	34 (25.2%)
	P-value	0.1944			0.4614		
Antibiotics are essential for managing the symptoms in children with COVID-19?	Correct (n=175)	54 (40.0%)	63 (31.3%)	58 (35.4%)	54 (47.0%)	79 (31.6%)	42 (31.1%)
	Incorrect (n=325)	81 (60.0%)	138 (68.7%)	106 (64.6%)	61 (53.0%)	171 (68.4%)	93 (68.9%)
	P-value	0.2626			0.0091		
Without the use of antibiotics, children usually suffer from greater periods of sickness?	Correct (n=195)	58 (43.0%)	76 (37.8%)	61 (37.2%)	46 (40.0%)	96 (38.4%)	53 (39.3%)
	Incorrect (n=305)	77 (57.0%)	125 (62.2%)	103 (62.8%)	69 (60.0%)	154 (61.6%)	82 (60.7%)
	P-value	0.5392			0.9560		
Not using antibiotics will prove beneficial for the children suffering from COVID-19?	Correct (n=90)	20 (14.8%)	36 (17.9%)	34 (20.7%)	22 (19.1%)	43 (17.2%)	25 (18.5%)
	Incorrect (n=410)	115 (85.2%)	165 (82.1%)	130 (79.3%)	93 (80.9%)	207 (82.8%)	110 (81.5%)
	P-value	0.4152			0.8902		
How often are antibiotics being prescribed to the COVID-19 children?	Correct (n=360)	102 (75.6%)	144 (71.6%)	114 (69.5%)	92 (80.0%)	171 (68.4%)	97 (71.9%)
	Incorrect (n=140)	33 (24.4%)	57 (28.4%)	50 (30.5%)	23 (20.0%)	79 (31.6%)	38 (28.1%)
	P-value	0.5059			0.0721		

## Discussion

The majority of parents surveyed in the study had a good understanding of the causes of viral infections and the fact that antibiotics were not necessary for treating them [19]. However, a significant proportion of parents still believed that antibiotics were essential for bacterial infections and for certain symptoms, such as earaches and runny noses [20]. Interestingly, many parents believed that antibiotics are always essential for children having symptoms of tonsillitis and otitis, despite the fact that antibiotics are not always necessary for these conditions [15]. Additionally, while most parents understood the importance of responsible antibiotic use and the risks of antibiotic resistance, many did not believe that antibiotics play any role in protection from the difficulties of the common flu or cold [21].

Overall, the study suggested that while many parents have a respectable and precise understanding of the appropriate use of antibiotics, there is still a need for education and awareness-raising on this topic. The fact that a substantial proportion of parents continue to believe that antibiotics are always required for certain ailments emphasises the significance of continued efforts to promote the safe use of antibiotics in order to combat antibiotic resistance [22]. A study conducted in China in 2020 assessed parental practices, attitudes, and knowledge on antibiotics use for childhood URTIs during the COVID-19 pandemic [23]. The study found that 29% of parents believed that antibiotics were effective in treating viral infections, and 65% believed that antibiotics were necessary for URTIs with fever. Moreover, 20% of parents reported stockpiling antibiotics at home, and 35% had administered antibiotics to their children without consulting a healthcare provider [24].

The study also found that parents who got information about antibiotic use from healthcare providers were more likely to have the right attitudes, knowledge, and practices about using antibiotics for URTIs [25]. This highlights the importance of providing accurate and consistent information to parents regarding the use of appropriate antibiotics for URTIs in children, especially during the COVID-19 pandemic [26]. Interventions such as educational campaigns, public health messages, and physician education could help promote appropriate antibiotic use for childhood URTIs during the COVID-19 pandemic [27]. These initiatives could educate parents on the significance of consulting a doctor before giving antibiotics to their children and help them recognise the warning signs of antibiotic resistance [28].

The present study showed that the female gender, increasing age and low socio-economic status were largely linked with poor parental attitude, knowledge, and practices regarding the usage of antibiotics for URTIs in children during the COVID-19 epidemic. Many of the women in our society remain at home and are housewives and this analysis shows that they did not get enough information about the utilisation of antibiotics for URTIs during the COVID-19 pandemic. A study from Syria analysing knowledge of antibiotic usage during COVID-19 found that there was a statistically significant difference between males and females. They noted that males had better knowledge about antibiotic usage during COVID-19 (p=0.023) [29]. This data raises the need for a better awareness campaign through print, electronic and digital media during a pandemic. Other researchers have also shown that poor knowledge about the COVID-19 pandemic has been shown to have an association with demographic and socioeconomic factors of individuals as was shown in the present research [30].

The study highlights the need for targeted educational campaigns for decreasing the misconceptions related to the use of antibiotics and increase awareness of the inappropriate risks, particularly in the case of high-risk groups. It also emphasises the importance of continuous training for paediatricians in promoting effective communication with parents regarding the prudent prescription of antibiotics. Targeted awareness of the particulars of COVID-19 is essential for the understanding of parents. It also suggests that pre- and post-training assessments for both parents and paediatricians to measure the effectiveness of education and training programs during endemics should be held. Additionally, some determinants are country-specific, representing the misuse of antibiotics such as particularities of the health care system, which should be assessed for developing multilevel programs of invention with the aim of limiting the spread of antibiotic resistance. Some interventions and activities such as antimicrobial stewardship should not be missed during the COVID-19 pandemic, thereby emphasising the importance of responsible antibiotic use to combat antimicrobial resistance.

Limitations of the study

The period of this study was relatively short and as it was a single-centre study, our findings cannot be generalised.

## Conclusions

Parental attitude, knowledge, and practices regarding the usage of antibiotics for URTIs in children during the COVID-19 pandemic showed variations. Parental attitude, knowledge and practices were associated with gender, age, and socio-economic status. Educational campaigns for the improvement of parental attitude, knowledge, and practices regarding the usage of antibiotics among children during viral infectious pandemics could play a vital role in reducing the inappropriate usage.

## References

[1] de Souza TH, Nadal JA, Nogueira RJ, Pereira RM, Brandão MB (2020). Clinical manifestations of children with COVID-19: A systematic review. Pediatr Pulmonol.

[2] Ye Q, Liu H (2022). Impact of non-pharmaceutical interventions during the COVID-19 pandemic on common childhood respiratory viruses-an epidemiological study based on hospital data. Microbes Infect.

[3] Panagakou SG, Spyridis N, Papaevangelou V (2011). Antibiotic use for upper respiratory tract infections in children: a cross-sectional survey of knowledge, attitudes, and practices (KAP) of parents in Greece. BMC Pediatr.

[4] Zhou J, Zhao P, Nie M, Gao K, Yang J, Sun J (2023). Changes of Haemophilus influenzae infection in children before and after the COVID-19 pandemic, Henan, China. J Infect.

[5] Kadambari S, Goldacre R, Morris E, Goldacre MJ, Pollard AJ (2022). Indirect effects of the covid-19 pandemic on childhood infection in England: population based observational study. Br Med J.

[6] Mameli C, Picca M, Buzzetti R (2022). Incidence of acute respiratory infections in preschool children in an outpatient setting before and during Covid-19 pandemic in Lombardy Region, Italy. Ital J Pediatr.

[7] Papadopoulos NG, Mathioudakis AG, Custovic A (2021). Childhood asthma outcomes during the COVID-19 pandemic: findings from the PeARL multi-national cohort. Allergy.

[8] Cohen R, Ashman M, Taha MK (2021). Pediatric Infectious Disease Group (GPIP) position paper on the immune debt of the COVID-19 pandemic in childhood, how can we fill the immunity gap?. Infect Dis Now.

[9] Mohebi L, Karami H, Mirsalehi N (2022). A delayed resurgence of respiratory syncytial virus (RSV) during the COVID-19 pandemic: an unpredictable outbreak in a small proportion of children in the Southwest of Iran, April 2022. J Med Virol.

[10] Oikonomou ME, Gkentzi D, Karatza A (2021). Parental knowledge, attitude, and practices on antibiotic use for childhood upper respiratory tract infections during COVID-19 pandemic in Greece. Antibiotics.

[11] Zhu Y, Li W, Yang B (2021). Epidemiological and virological characteristics of respiratory tract infections in children during COVID-19 outbreak. BMC Pediatr.

[12] Toth JM, Rosenthal M, Sharma M, Barnard M (2021). Caregivers’ perspectives of pharmacist intervention in children’s antibiotic prescriptions for upper respiratory tract infections. J Pharm Pract.

[13] Ye Q, Wang D (2022). Epidemiological changes of common respiratory viruses in children during the COVID-19 pandemic. J Med Virol.

[14] Mijović B, Aćimović J, Dević JD (2022). Knowledge, attitudes and practices of parents and pediatricians regarding antibiotic use among children: differences in relation to the level of education of the parents in the Republic of Srpska Bosnia and Herzegovina. Antibiotics.

[15] Korppi M, Heikkilä P, Palmu S, Huhtala H, Csonka P (2022). Antibiotic prescribing for children with upper respiratory tract infection: a Finnish nationwide 7-year observational study. Eur J Pediatr.

[16] Katz SE, Spencer P, Cates J, Harnack L, Xu M, Banerjee R (2022). Improvements in appropriate ambulatory antibiotic prescribing using a bundled antibiotic stewardship intervention in general pediatrics practices. Infect Control Hosp Epidemiol.

[17] Uppala R, Sitthikarnkha P, Niamsanit S (2022). Effect of the COVID-19 pandemic on lower respiratory tract infection determinants in Thai hospitalized children: national data analysis 2015-2020. Trop Med Infect Dis.

[18] Kelvin AA, Halperin S (2020). COVID-19 in children: the link in the transmission chain. Lancet Infect Dis.

[19] Torretta S, Capaccio P, Coro I (2021). Incidental lowering of otitis-media complaints in otitis-prone children during COVID-19 pandemic: not all evil comes to hurt. Eur J Pediatr.

[20] Kaur R, Schulz S, Fuji N, Pichichero M (2021). COVID-19 Pandemic Impact on Respiratory Infectious Diseases in Primary Care Practice in Children. Front Pediatr.

[21] Diesner-Treiber SC, Voitl P, Voitl JJ (2021). Respiratory infections in children during a Covid-19 pandemic winter. Front Pediatr.

[22] She J, Liu L, Liu W (2020). COVID-19 epidemic: disease characteristics in children. J Med Virol.

[23] Jia R, Lu L, Su L (2022). Resurgence of respiratory syncytial virus infection during COVID-19 pandemic among children in Shanghai, China. Front Microbiol.

[24] Mullish BH, Marchesi JR, McDonald JA (2021). Probiotics reduce self-reported symptoms of upper respiratory tract infection in overweight and obese adults: should we be considering probiotics during viral pandemics?. Gut Microbes.

[25] Ippolito G, La Vecchia A, Umbrello G (2021). Disappearance of seasonal respiratory viruses in children under two years old during COVID-19 pandemic: a monocentric retrospective study in Milan, Italy. Front Pediatr.

[26] Pines JM, Zocchi MS, Black BS, Carlson JN, Celedon P, Moghtaderi A, Venkat A (2021). Characterizing pediatric emergency department visits during the COVID-19 pandemic. Am J Emerg Med.

[27] Radhakrishnan L, Carey K, Hartnett KP (2022). Pediatric emergency department visits before and during the COVID-19 pandemic — United States, January 2019-January 2022. MMWR Morb Mortal Wkly Rep.

[28] Vierucci F, Bacci C, Mucaria C, Dini F, Federico G, Maielli M, Vaccaro A (2020). How COVID-19 pandemic changed children and adolescents use of the emergency department: the experience of a secondary care pediatric unit in central Italy. SN Compr Clin Med.

[29] Swed S, Shoib S, Almoshantaf MB (2022). Knowledge, attitudes, and practices related to COVID-19 infection, related behavior, antibiotics usage, and resistance among Syrian population: a cross-sectional study. Health Sci Rep.

[30] Rabbani MG, Akter O, Hasan MZ, Samad N, Mahmood SS, Joarder T (2021). COVID-19 knowledge, attitudes, and practices among people in Bangladesh: telephone-based cross-sectional survey. JMIR Form Res.

